# Gut Dysbiosis and Western Diet in the Pathogenesis of Essential Arterial Hypertension: A Narrative Review

**DOI:** 10.3390/nu13041162

**Published:** 2021-04-01

**Authors:** Maria Paola Canale, Annalisa Noce, Manuela Di Lauro, Giulia Marrone, Maria Cantelmo, Carmine Cardillo, Massimo Federici, Nicola Di Daniele, Manfredi Tesauro

**Affiliations:** 1 Department of Systems Medicine, University of Rome Tor Vergata, Via Montpellier 1, 00133 Rome, Italy; canale@uniroma2.it (M.P.C.); federicm@uniroma2.it (M.F.); 2 UOC of Internal Medicine-Center of Hypertension and Nephrology Unit, Department of Systems Medicine, University of Rome Tor Vergata, Via Montpellier 1, 00133 Rome, Italy; dilauromanuela@gmail.com (M.D.L.); giul.marr@gmail.com (G.M.); didaniele@med.uniroma2.it (N.D.D.); 3 PhD School of Applied Medical, Surgical Sciences, University of Rome Tor Vergata, Via Montpellier 1, 00133 Rome, Italy; 4 School of Specialization in Geriatrics, University of Rome Tor Vergata, 00133 Rome, Italy; mariacantelmo90@gmail.com; 5 Department of Internal Medicine and Geriatrics, Policlinico A. Gemelli IRCCS, 00168 Roma, Italy; carmine.cardillo@unicatt.it

**Keywords:** arterial hypertension, gut microbiota, Western diet, high salt-intake diet, prebiotic, probiotic, fecal transplant

## Abstract

Metabolic syndrome is a cluster of the most dangerous cardiovascular (CV) risk factors including visceral obesity, insulin resistance, hyperglycemia, alterations in lipid metabolism and arterial hypertension (AH). In particular, AH plays a key role in the complications associated with metabolic syndrome. High salt intake is a well-known risk factor for AH and CV diseases. Vasoconstriction, impaired vasodilation, extracellular volume expansion, inflammation, and an increased sympathetic nervous system (SNS) activity are the mechanisms involved in the pathogenesis of AH, induced by Western diet. Gut dysbiosis in AH is associated with reduction of short chain fatty acid-producing bacteria: acetate, butyrate and propionate, which activate different pathways, causing vasoconstriction, impaired vasodilation, salt and water retention and a consequent high blood pressure. Moreover, increased trimethylamine N-oxide and lipopolysaccharides trigger chronic inflammation, which contributes to endothelial dysfunction and target organs damage. Additionally, a high salt-intake diet impacts negatively on gut microbiota composition. A bidirectional neuronal pathway determines the “brain–gut” axis, which, in turn, influences blood pressure levels. Then, we discuss the possible adjuvant novel treatments related to gut microbiota modulation for AH control.

## 1. Introduction

Arterial hypertension (AH) is one of the most important constituents of metabolic syndrome (MetS) and represents a global health problem with implications for public health costs and healthcare personnel involved for its treatment. AH is divided into two forms: primary or essential and secondary. In the first one, the rise of blood pressure (BP) is due to undetermined causes and it represents about 90% of the cases, while in the second one, it is possible to determine a specific cause [1,2].

AH is a potent modifiable cardiovascular (CV) risk factor and is the leading cause of mortality related to chronic kidney failure, ischemic heart disease and stroke [3,4,5,6]. According to ATP III criteria, MetS is characterized by at least three of the following five parameters: glycemia >100 mg/dL or glucose-lowering therapy, high-density lipoprotein- cholesterol (HDL-C) <40 mg/dL (men) or HDL-C <50 mg/dL (women) or lipid-lowering therapy, triglycerides (TG) >150 mg/dL, waist circumference (WC) >102 cm (men) or >88 cm (women), BP > 130/85 mmHg or anti-hypertensive therapy [7]. There are several possible mechanisms which explain the AH develop in the MetS. Among these, obesity and the accumulation of visceral adipose tissue, insulin resistance, low-grade inflammation, endothelial dysfunction, oxidative stress, dyslipidemia, sympathetic overactivity, activated renin-angiotensin aldosterone system (RAAS) certainly play an important role [8,9,10,11,12,13,14].

In the 2018 European Society of Cardiology (ESC)/ European Society of Hypertension (ESH) guidelines for the management of AH recommend “to lower BP to <140/90 mmHg in all patients and, if treatment is well tolerated, BP values should be targeted to 130/80 mmHg or lower in most patients. Moreover, in patients <65 years it is recommended that systolic blood pressure (SBP) should be lowered to BP range of 120–129 mmHg in most patients” [15]. American Heart Association (AHA) and the American College of Cardiology (ACC) redefined AH as “a BP at or above 130/80 mm Hg”, lowering the cut-off for the AH diagnosis. According to this new cut-off, half of the American US adult population falls in the group of hypertensive subjects [16]. Moreover, the prevalence of AH is expected to increase in the following years and to reach 1.56 billion people in the world by 2025 [17]. Despite extensive research on AH and the wide availability of drugs with different mechanisms of action to be used alone or in combination, BP control is sometimes insufficient. Furthermore, from 10 to 30% of patients are affected by resistant AH, defined as BP control not achieved by three anti-hypertensive drugs, from different classes, one of which should be a diuretic [18]. Dietary recommendations are part of non-pharmacological treatment, according to international guidelines, also for patients requiring pharmacological treatment [18]. Despite treatment, just half of AH patients reach target values [19].

Most of the immune and microbial cells in the body reside within the gastrointestinal tract [20]. Intestinal microbes, known as “gut microbiota”, have the ability to modulate the physiological functions of the body by stimulating the immune system and favoring the extraction of energy and vitamins from ingested foods [21]. Gut microbiota is made up of bacteria, archaea, fungi, protozoa and viruses [22] and its different microbial communities interact with each other, affecting the host health. The fibers introduced with the diet are digested by the gut microbiota but not completely hydrolyzed and are metabolized and used by the commensal bacteria of the colon as a source of energy. This phenomenon causes the production of intestinal metabolites, called short-chain fatty acids (SCFAs), like acetate, propionate and butyrate. There are numerous studies that link these metabolites with the microbiota, the epithelium and the immune system [23]. In the case of gut structural alterations associated with a change in the quali-quantitative composition of the microbiota, it is possible to define the condition of “gut dysbiosis”. It is associated with chronic intestinal inflammatory and chronic degenerative non-communicable diseases (CNCDs). In fact, over the past years alterations in gut microbiota have been found in different CNCDs [24,25,26,27,28,29]. Recently, gut dysbiosis has also been linked to AH [30,31].

High salt intake is a well-known risk factor for AH and CV diseases [17,32]. At gut level, sodium interacts with commensal microbiota and can lead to AH, as recently explored by several authors [4,33,34].

In this review, we summarize the recent and current literature to further understand the role of Western diet and gut dysbiosis in the pathogenesis and progression of essential AH. Finally, we discuss the possible adjuvant novel treatments related to gut microbiota modulation for the AH control.

## 2. Impact of Western Diet on Arterial Hypertension

Essential AH results from the interplay of genetic, environmental and behavioral factors. Among environmental and behavioral factors, dietary habits related to geographical area, culture and ethnic group play an important role [29,35,36]. Essential AH is absent in populations with very low daily sodium intake [37]. In developed countries, diets based on processed foods are high in sodium and low in potassium [37]. In the late 80s, INTERSALT Study showed a positive correlation between sodium intake and BP values [38]. Later, the dietary approaches to stop hypertension (DASH)-Sodium Collaborative Research Group evaluated the effects of different sodium intake in combination with the DASH diet (rich in fruit, vegetables and low-fat dairy products) on BP control, demonstrating its efficacy in counteracting AH [39]. It is worth noting that in the DASH diet, the potassium content was twice as high with respect to the typical American diet. This observation suggests that a lower sodium to potassium ratio is involved in BP reduction. In particular, the authors speculated that it is important not only to reduce the sodium intake but also to increase potassium intake [40]. Additionally, in the INTERSALT study 24h-potassium excretion was inversely related to SBP and diastolic blood pressure (DBP) [38]. Interestingly, in the induced-hypertensive mice model, after the administration of deoxycorticosterone acetate (DOCA)/salt, the dietary potassium supplementation was able to lowered BP values but also to ameliorate end-organ damage, preventing cardiac hypertrophy [41,42]. Later, a meta-analysis of 33 randomized clinical trials confirmed the possible role of potassium supplementation in lowering BP [43]. Moreover, dietary potassium was able to inhibit sodium sensitivity in hypertensive and normotensive subjects [44].

## 3. Pathophysiological Mechanisms of Western Diet Leading to Arterial Hypertension

**Figure 1:**
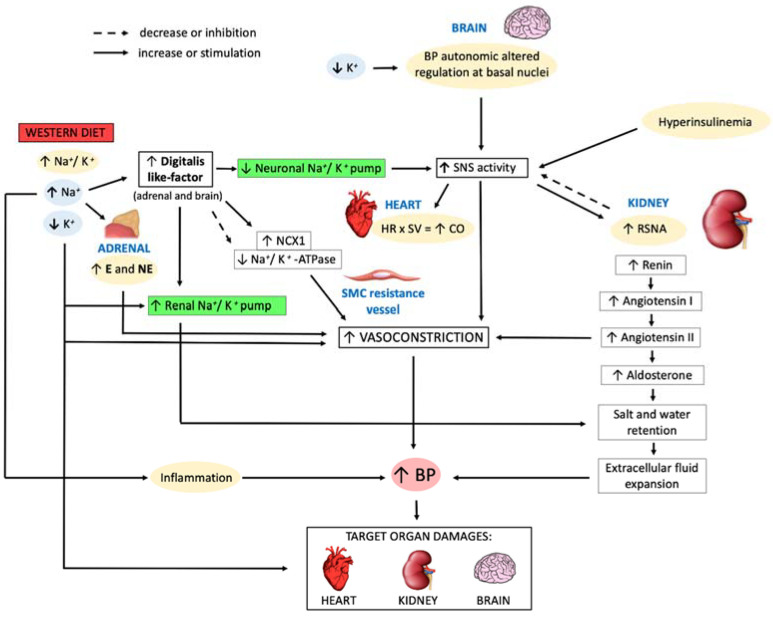
Western diet contribution to pathogenesis of essential arterial hypertension. Abbreviations: BP, blood pressure; CO, cardiac output; E, epinephrine; HR, heart rate; NCX1, sodium-calcium exchanger type 1; NE, norepinephrine; RSNA, renal sympathetic nerve activity; SMC, smooth muscle cell; SNS, sympathetic nervous system; SV, stroke volume.

Figure 1 presents the main pathophysiological mechanisms underlying Western diet-related AH, which will be further discussed in this and in the following paragraphs.

The modern Western diet is characterized by low potassium and a high sodium intake, that contributes to inducing AH through different mechanisms. In fact, an excessive sodium intake in the body determines extracellular fluids expansion leading to release of digitalis-like factor (digitalis-LF) from the adrenal gland and the brain [37]. Digitalis-LF inhibits the sodium pump present in different organs such as arterial wall smooth-muscle cells (SMCs). At arterial wall SMCs, sodium and digitalis-LF high concentrations result in sodium pump inhibition with consequent stimulation of the sodium–calcium exchanger type 1 (NCX1) and an increase in calcium intracellular concentration which determines vascular contraction [45]. Moreover, digitalis-LF inhibits Na^+^/K^+^-ATPase pump present in arterial wall SMCs, induces vasoconstriction and, therefore, increases BP [46]. Low potassium not only acts on the sodium pump, but also inhibits potassium channels in the cell membrane, promoting an increase in intracellular calcium and the consequent contraction of arterial wall SMCs. In fact, their contraction, as previously described, enhances peripheral vascular resistances causing BP increase. At renal level, in response to a high salt-intake diet, the circulating digitalis-LF increases both the activity and the expression of the renal sodium pump, determining sodium retention [37,47]. Potassium depletion stimulates the activity and expression of the renal sodium pump, thus promoting sodium retention [47,48].

Additionally, sodium excess inhibits endothelium-dependent vasodilation by a double mechanism: decreasing nitric oxide (NO) synthesis and increasing plasma levels of the endogenous inhibitor of NO, such as asymmetric dimethylarginine (ADMA) [49]. Indeed, a high-potassium diet causes endothelium-dependent vasodilation [49]. Moreover, in a rat hypertensive model, Gradin et al. demonstrated that a high-sodium diet induced an increase of BP values through another mechanism, namely an enhancement of plasma norepinephrine (NE) levels [50]. Changes in the activity both of the neuronal sodium pump in the brain and of the RAAS occur following modifications of sodium and potassium levels in the cerebrospinal fluid and in the bloodstream [37,51,52]. These changes determine an increase in central sympathetic outflow, resulting in BP enhancement [53,54].

Moreover, another cause of AH seems to be the increase in renal sympathetic nerve activity (RSNA), induced in turn, by an enhanced sympathetic nerve system (SNS) activity. In fact, enhanced RSNA lead to an increase of BP through three different ways: (i) increase of tubular reabsorption of urinary sodium and water, (ii) reduction of renal blood flow that, in turn, induces low glomerular filtration rate, (iii) renin release by juxtaglomerular apparatus with RAAS activation [55]. At the cardiac level, the enhanced SNS activity induces high heart rate and stroke volume, which determines an increase in cardiac output [56].

Aside from the above-mentioned effects on BP, in the early 90s, an experimental animal study showed the effects of a high-potassium diet on the reduction of atherosclerotic plaque deposition in aortas of hypercholesterolemic hypertensive rats [57]. Additionally, an in vitro study demonstrated that potassium is able to inhibited cultured vascular SMCs proliferation [58]. Moreover, potassium inhibited free radical formation by cell lines derived from endothelium and from monocytes/macrophages [59]. In recent years, it has been demonstrated that reduced dietary potassium intake is capable of inducing the vascular calcification, increasing aortic stiffness in ApoE-deficient mice, compared to mice fed with a normal content potassium diet. On the contrary, a higher dietary potassium intake ameliorated vascular calcification and aortic stiffness. The author hypothesized that the decrease of potassium levels induces an enhancement of intracellular calcium. The latter is able to activate a cAMP response element-binding protein (CREB) signal, causing the calcification of vascular SMCs [60]. These studies suggest that the role of potassium goes beyond the BP reduction, counteracting the target organs damage, thus impacting on overall CV risk.

Interestingly, high dietary sodium intake contributes to AH also triggering inflammation. Studies conducted in the last decade have demonstrated how a high dietary salt intake increases interleukin (IL)-17 that causes enhanced BP levels [61,62,63]. In particular, after the infiltration of perivascular and renal space, in response to hypertensive stimuli, T cells produce pro-inflammatory cytokines that determine vascular and renal interstitial dysfunction, resulting in a BP increase [64,65,66].

In a recent paper, Barbaro et al. demonstrated that salt enters in dendritic cells through amiloride-sensitive transporters and activates reactions that lead to reactive oxygen species (ROS) production and to the formation of isolevuglandins (IsoLG)-protein adducts [33]. Then, IsoLGs promote T cells activation and induces AH [67].

Therefore, at the present time, literature evidences suggest that Western diet contributes to the AH development through different mechanisms: vasoconstriction, impaired vasodilation, extracellular volume expansion, inflammation, and increased SNS activity [68].

## 4. Gut Microbiota Qualitative and Quantitative Changes in Arterial Hypertension

Gut microbiota composition is characterized by bacterial members of the Firmicutes and Bacteroidetes phyla [69]. The term “dysbiosis” refers to the qualitative and quantitative gut microbiota alterations. Both in animal models and human, AH was associated with gut dysbiosis characterized by higher Firmicutes (F) to Bacteroidetes (B) ratio, α-diversity reduction, decreased richness, increase in lactate producing bacteria, and decrease in acetate- and butyrate-producing bacteria [30]. In an interesting study, Li et al. found no differences in the gut microbiota composition of pre-hypertensive and hypertensive patients compared to normotensive subjects [31]. Mice models confirmed that the gut microbiota dysbiosis causes AH and cardiac hypertrophy, explaining the possible mechanisms. Among these, systemic inflammation, induced by angiotensin II, plays a pivotal role [70,71]. A recent contribution from Robles-Vera et al. summarizes the characteristics of gut dysbiosis in AH experimental models [19]. In order to assess a cause–effect relationship, researchers transplanted microbioma from hypertensive animal models or hypertensive patients to normotensive animal recipients (Table 1). The normotensive animal recipients developed AH, demonstrating the contribution of gut dysbiosis in the AH pathogenesis.

An extensive review recently published from Verhaar et al. analyzed gut microbiota composition in humans [72]. The authors reported the association between higher BP values and lower gut microbiota α-diversity in almost all studies [30,31,73,74,75,76]. It is worth noting that low α-diversity is also present in dyslipidemia, hyperinsulinemia and obesity [9,72]. SCFAs-producing bacteria decrease in hypertensive patients compared to normotensive subjects and these bacteria are obtained by the fermentation of indigestible dietary fibers [31,74,77,78,79]. Acetate and propionate are absorbed by the gut and enter in the bloodstream, whereas butyrate is only absorbed in small amounts [72]. Hypertensive patients also showed an increase in microbiota Gram-negative species, with consequent increase of lipopolysaccharides (LPS) [72].

## 5. Pathophysiological Mechanisms in Gut Dysbiosis Leading to Arterial Hypertension

**Figure 2:**
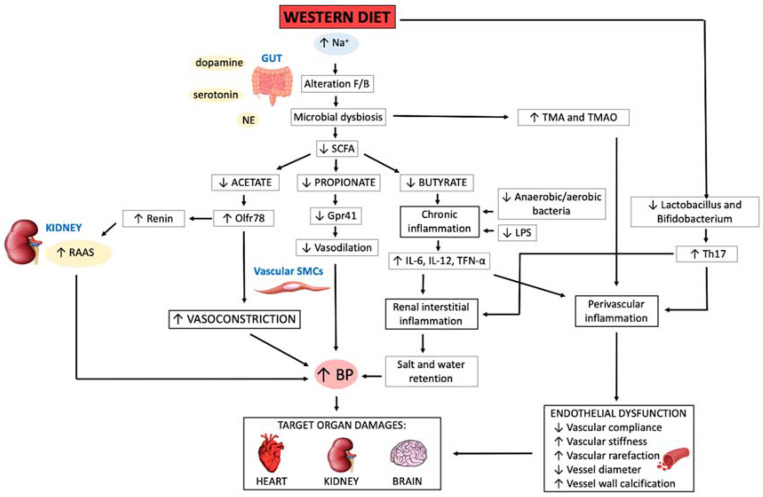
Gut dysbiosis contribution to the pathogenesis of essential arterial hypertension. Abbreviations: B, Bacteroidetes; BP, blood pressure; F, Firmicutes; Olfr78, olfactory receptor 78; Gpr41, G protein-couple receptors 41; IL, interleukin; LPS, lipopolysaccharide; NE, norepinephrine; RAAS, renin angiotensin-aldosterone system; SCFA, short chain fatty acids; SMC, smooth muscle cell; TMA, trimethylamine; TMAO, trimethylamine N-oxide; TNF, tumor necrosis factor.

The main pathophysiological mechanisms involved in gut dysbiosis that lead to AH are shown in Figure 2. The gut microbiota can modulate secretion of different hormones such as NE, dopamine and serotonin which enter in the bloodstream and thereby affect the BP [3].

Western diet, characterized by a high dietary salt intake enhances the Firmicutes to Bacteroidetes ratio, mechanism at the base of gut dysbiosis. SCFAs, end-products of fermentation of dietary fibers produced by the gut microbiota anaerobes, are absorbed at colonic level and enter in the bloodstream. Dysbiosis leads to a decrease of SCFAs production, namely a decrease in acetate, propionate and butyrate. A growing body of evidences indicates that gut microbiota influences BP also through SCFAs production. An important study by Pluznick et al., showed that SCFAs produced by the gut microbiota, and particularly acetate and propionate, modulate BP via two SCFAs receptors, namely olfactory receptor 78 (Olfr78) and G protein-coupled receptor (Gpr)41 [85]. Olfr78 is expressed in SMCs of arterial walls. In particular, this receptor in the glomerular afferent arteriole plays an important role in renin secretion, while in arterial wall SMCs it mediates the vasoconstriction.

In wild-type mice, propionate causes vasodilation resulting in acute hypotension. This animal study indicates that Gpr41 contributes to the hypotensive effects of propionate, whereas Olfr78 functions raises BP and antagonize the hypotensive effects of propionate. Interestingly, Pluznick et al. also demonstrated that, in Olfr78 null mice, antibiotic administration influenced BP, confirming that SCFAs contribute to BP regulation [86]. Additionally, SCFAs act on the intestinal cells and also on local immune system. Moreover, acetate is able to activate Gpr43 (another G-protein-coupled receptor) in neutrophils present in the lamina propria [19]. Butyrate was shown to attenuate the pro-inflammatory state consequent to LPS-stimulation [72,87]. In a recent contribution from Robles-Vera et al., the authors demonstrated that chronic oral acetate/butyrate administration prevented the AH onset in spontaneously hypertensive rat (SHR) [19].

Further animal model studies demonstrated that a high-salt diet induces a decrease in Lactobacillus and Bifidobacterium of the gut microbiota [88]. A study conducted by Miranda et al. showed that the reduction of Lactobacillus causes an enhancement in T helper-17 (Th17), which, in turn, characterizes the inflammatory status through the release of pro-inflammatory cytokines [89]. Th17 cells are localized mainly at the perivascular level and their impaired activation is responsible for the endothelial dysfunction, associated with heart, kidney and brain damage [90].

Gut microbiota also exerts its influence through other bacterial metabolic substances as Trimethyilamine N-oxide (TMAO) [19,91]. Trimethylamine (TMA) results from the metabolism of trimethylammonium-containing foods as meat and eggs, and in the liver is converted into TMAO [72]. TMAO may impact on BP regulation, enhancing pro-inflammatory cytokines (like tumor necrosis factor -TNF-α, interleukin- IL-1β) and decreasing anti-inflammatory cytokines (such as IL-10) release [72,92]. Although a direct role of TMAO in determining atherosclerosis has not been proven, its levels are associated with an increase in CV risk and in all-cause mortality [72,93,94,95].

Additionally, gut microbiota acts through bacterial wall components as LPS, and its high circulating levels result from increased gut permeability [19,96,97]. Moreover, LPS increase determines chronic inflammation through the production of pro-inflammatory cytokines, such as TNF- α, IL-12, and IL-6 [72].

Overall, from the current understanding, a decrease in SCFAs secondary to gut dysbiosis may be interlaced to BP regulation and target organs damage through a variety of pathophysiological mechanisms: direct and indirect vasoconstriction, impaired vasodilation, extracellular volume expansion secondary to both an increased renin secretion and a consequent RAAS activation. All these alterations entail aldosterone production, endothelial dysfunction, systemic and renal interstitial inflammation [68].

## 6. Gut Microbiota, Western Diet and Sympathetic Nervous System Interactions

Although gut microbiota transplantation studies suggest a cause–effect relationship between gut dysbiosis and essential AH, it is very important to remind the existence of bidirectional neural pathways from the gut to the paraventricular nucleus in the brain. SNS overactivity increases gut inflammation and permeability leading to dysbiosis, which, in turn, increases BP through the above-mentioned mechanisms [98,99]. In the early 70s, Furness et al. illustrated the influence of SNS on gastrointestinal secretion and absorption and on gastrointestinal blood flow [100]. More recently, Zubcevic et al. showed that neuroinflammation in the hypothalamic area causes an enhancement in SNS activity that, in turn, modulates the gut microbiota composition in angiotensin II-induced AH model [101]. The results of this study indicate that the presence of an impaired autonomic nervous system response plays a role in gut dysbiosis. Interestingly, Robles-Vera et al. demonstrated in SHR that losartan changes the gut microbiota composition, ameliorating gut dysbiosis through the reduction of F to B ratio. Moreover, the drug improves the gut barrier integrity and restores colonic α-defensins production, actions that contribute to its anti-hypertensive effects [102].

The enteric nervous system communicates with the nucleus of the solitary tract in the brain through the vagal nerve. Animal studies have suggested that high colonic acetate levels may lower BP through parasympathetic activation [72]. Moreover, a recent study demonstrated that butyric acid can exert hemodynamics effects in terms of decreasing BP. In fact, its intravenous administration causes a reduction in BP levels, but this effect is mediated by SNS, supporting the role of gut–brain axis in BP regulation. To prove this, the subphrenic vagotomy reduces the butyric acid hypotensive effects [103].

LPS administration increased NE levels, heart rate and neuroinflammation [72,104]. A study from Sandiego et al. provided insight into induced neuroinflammation by LPS administration in humans, showing brain microglial activation on positron emission tomography scans [105]. In turn, hypothalamic paraventricular nucleus inflammation triggers SNS activity from the brain to peripheral targets. Finally, it is worth stressing that the increase in sympathetic tone associated with obesity/hyperinsulinaemia [106].

Currently, these observations indicate that the association between gut dysbiosis, Western diet and BP regulation is an interplay rather than a cause–effect relationship.

Pathophysiological mechanisms presented in this review, added to “already known” neural, endocrine, renal and CV mechanisms, make the pathophysiology of AH even more complex. The multifactorial role of Western diet and gut dysbiosis in the pathogenesis of essential AH offers a potential new insight regarding non-pharmacological adjuvant treatment.

## 7. New Therapeutic Approaches in the Prevention and Treatment of Arterial Hypertension

Each individual presents a set of gut microorganisms that colonize the intestine and tend to remain stable throughout their entire lifespan [107,108]. However, transient short-term fluctuations in the gut microbiota have recently been observed [109].

Alterations in the gut microbiota can modify intestinal homeostasis and the immune system, causing diseases onset such as MetS, obesity, inflammatory bowel diseases, diabetes mellitus, neurodegenerative diseases etc. [71,110,111]. One of the environmental factors able to modify its composition is represented by dietary habits. The gut microbiota appears to have a different composition in various people due to diet differences, as different diets would seem to influence its composition, contributing to its diversity. Unfortunately, it is currently unknown whether long-term dietary intervention impact gut microbiota changes, due to the lack of controlled clinical trials [112,113]. The composition of the diet, in fact, amply modulates the various gut microbial species and, therefore, it seems to play an important role in the prevention and management of CNCDs, in particular of AH [28,29,114,115]. Thus, nutritional treatments useful in modulating the gut microbiota are at the center of the interest of the scientific community, although it is not known whether they are able to permanently change its composition [116].

The type and amount of proteins, fats and carbohydrates present in the diet has been widely demonstrated to influence the composition of the host’s gut microbiota. High-carbohydrate diets, for example, favors the Prevotella genus while, high-fat and high-protein diets promote the development of Bacteroidetes microbial species. This effect is related to the metabolites that derives by their digestion. Furthermore, it was observed that mice fed with high-fat and protein diets tended to have an increase in Lactobacillaceae and a decrease in Clostridiaceae species, compared to mice fed *ad libitum* [117]. In the gastrointestinal tract, some carbohydrates, such as starch and non-starch polysaccharides, cannot be metabolized directly by the host and are used as an energy resource for microbial growth. For such functions, these carbohydrates are also considered “prebiotics”. The prebiotics have been defined as “a substrate that is selectively used by host microorganisms conferring a health benefit” [118]. Prebiotics are able to induce variations in the composition of the microbiota and have multiple beneficial effects for the health of the host. Prebiotics such as inulin, fructo-oligosaccharides and oligosaccharides act as growth stimulators of Bifidobacteria, Lactobacilli and lactic acid bacteria [119]. The gut microbiota is involved in the regulation of antimicrobial peptides, in the gene expression of mucin, and in the paracellular permeability of small intestine Paneth cells. Consequently, a healthy microbiota is able to positively modulate the immune system by stimulating the maturation of B and T lymphocytes [120].

Probiotics have been defined as “live microorganisms which, when administered in adequate amounts, confer a health benefit on the host” [121]. Traditionally probiotics were used in the fermentation of food and currently they are also consumed by subjects as oral supplements. In fact, probiotics are able to colonize and proliferate within the gastrointestinal system, influencing its homeostasis and modifying the production of beneficial metabolites derived from fermentation. Numerous studies have shown that probiotics are able to help in the treatment of diarrhea associated with infections or prolonged use of antibiotics, of glycometabolic control in diabetic patients, and of chronic inflammatory bowel diseases [122,123]. However, it must be emphasized that the capabilities of probiotics can be highly unpredictable and change from subject to subject [124,125]. Further action of probiotics, in addition to promoting intestinal health, seems to be the reduction of BP levels, both SPB and DBP [126]. The combined use of probiotics with anti-hypertensive drugs should be associated with a healthy diet and lifestyle changes. In fact, a healthy diet—characterized by low sodium intake, high fruit and vegetables intake, low in sweets, meat and saturated fatty acids and with a moderate alcohol consumption—is useful in the treatment of AH [79]. Certainly, probiotics do not act as a drug, but as an adjuvant treatment to the traditional anti-hypertensive drugs, in particular their long-term assumption contributes to reduced BP values [127,128,129].

A study by Li J et al., described the link between high BP and gut dysbiosis [31]. This study was based on analysis of the microbiota in 41 healthy subjects, 56 pre-hypertensive subjects and 99 hypertensive patients. In the pre-hypertensive group, SPB levels were between 125–139 mm Hg, while DBP was between 80–89 mm Hg. In the hypertensive group, the SPB was greater than or equal to 140 mm Hg and the DBP greater than or equal to 90 mm Hg. In all enrolled subjects, total sequencing of bacterial DNA of fecal samples were identified. Pre-hypertensive and hypertensive patients presented an equal profile of the gut microbiota and a lesser diversity of bacteria than the group of healthy subjects. Gram-negative bacteria such as *Prevotella* and *Klebsiella* dominated over other strains, being producers of bacterial endotoxins with pro-inflammatory action [31], confirming that the alteration of gut microbiota contributes to the pathogenesis of AH. The restoration of gut microbiota homeostasis, condition called “eubiosis”, seems to be the better strategy to counteract the onset of AH.

**Figure 3:**
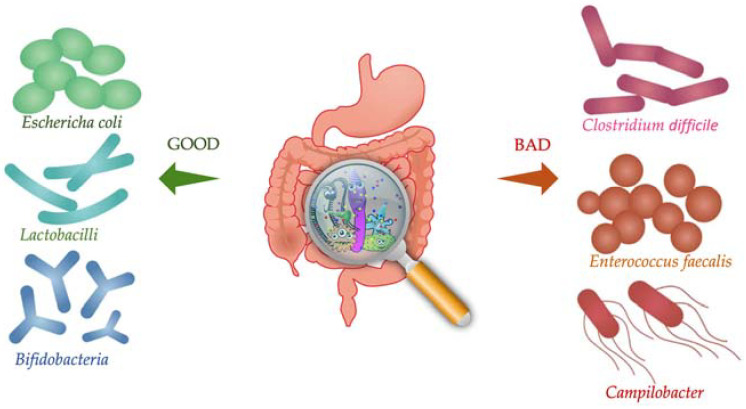
Effects of bacterial species on arterial hypertension.

In Figure 3, we summarized the most common bacterial species of gut microbiota that influence positively and negatively the onset and the control of AH.

In hypertensive patients, in addition to the prevalence of some strains with pro-inflammatory action, it has been observed a loss of bacteria involved in the synthesis and transport of amino acids essential such as lysine, histidine and serine, and in the metabolism of fatty acids, influencing, in turn, energy expenditure. Other effects of gut dysbiosis related to AH were increased kidney vasculature, inflammation of vascular tissue, and decreased production of NO [130].

As previously described, the association between gut microbiota and AH have been recently highlighted. Taking into consideration the experimental models of hypertension studied (SHR, Dahl salt-sensitive- SS, Ang II and DOCA- salt), it is possible to note the presence of an altered microbiota in all cases [30,82]. To determine the link between microbiota and AH, gnotobiotic animal models, without microbiota, were used. A study on transplanted fecal samples from one normotensive and two untreated hypertensive subjects into gnotobiotic mice, demonstrated that the gnotobiotic mice developed a gut microbiota similar to that of their donors [31]. Gnotobiotic mice that received transplantation from hypertensive subjects exhibited an altered microbiota with a reduced α-diversity and higher SPB and DPB levels than mice transplanted with stool sample from normotensive subjects.

Further studies showed that the microbiota and its metabolites were able to predict the development of AH. In fact, in hypertensive subjects, some opportunistic species like *Klebsiella* spp., *Streptococcus* spp. and *Parabacteroides merdae* were more represented compared to normotensive subjects [31,77].

The relationship between the intestinal microbiota, inflammation and BP appears to be different in the two sexes. Women of childbearing age have lower BP and arterial stiffness than men, but these parameters are higher in postmenopausal women [131]. In fact, epidemiologically, AH presents gender differences. The prevalence of AH is higher in men than in women up to 65 years of age, subsequently this prevalence becomes higher in women. One of the factors that can be related to these epidemiological changes is certainly menopause. It is hypothesized that a further mechanism involved in the AH epidemiological differences is the different gut microbiota composition among the two sexes [132]. In a large cohort of European subjects belonging to all age groups, it was highlighted that male subjects have a higher presence of *Bacteroides* and *Prevotella* than female subjects [133]. Furthermore, the possible relationship between the gut microbiota, sex hormones, and diet was examined in animal models. Specifically, gender differences between α- and β- diversity were highlighted [134]. In male rats, it was shown that sex hormones have a high impact on the microbiome regardless of diet, while in female rats this impact was evident only following a high-fat diet [135].

Differences gender-related in the gut microbiota composition appear to be predominantly induced by sex hormones. Indeed, gender differences in the gut microbiota are not evident until puberty. This data supports the hypothesis that sex hormones may play a key role in the shaping of the gut microbiota composition [136,137]. In particular, after puberty there is a difference in α-diversity as evidence by numerous animal studies, while this difference tends to disappear in the elderly [137,138].

SCFAs are essential for intestinal homeostasis and their absence could be involved in the pathogenesis of AH. In fact, both in the experimental models of hypertension and in hypertensive subjects, there is a reduction of the bacteria that produce the acetate and butyrate metabolites. In the mouse model with nDOCA salt, it was shown that fibers and acetate were able to modulate the gut microbiota and reduce BP values [139].

## 8. Conclusions

The complex interplay between Western diet, gut dysbiosis and essential AH opens the way to new adjuvant non-pharmacological therapeutic frontiers. However, it should be kept in mind that studies conducted so far highlight the pathophysiological mechanisms mainly in animal models. A further research is needed in humans to better identify the gut microbiota characteristics to develop new therapeutic strategies.

## Figures and Tables

**Table 1:** Fecal microbiota transplantation studies in hypertensive animals and humans.

Author	Donor	Recipient	Nutritional Treatment	Primary Outcome	*p*	Year
Mell et al. [80]	Dahl salt-resistant rats	Dahl salt-sensitive rats	high-salt diet	↑ BP ↓ Na^+^ excretion Shorter life span	<0.05 <0.01 <0.05	2015
Durgan et al. [81]	Hypertensive rats with OSA	Normotensive rats with OSA	Donor: high-fat diet Recipient: normal diet	↑ BP	<0.05	2016
Adnan et al. [82]	SH rats	Normotensive WKY rats	N.A.	↑ BP ↑ F to B ratio	0.02 0.042	2017
Li et al. [31]	Hypertensive subjects	Germ-free mice	N.A.	↑ systolic BP ↑ diastolic BP	0.018 0.019	2017
Toral et al. [83]	SH rats	Normotensive WKY rats	N.A.	↑ systolic BP ↑ diastolic BP ↑ plasma noradrenaline ↑ TNF-α, IL-1β, IL-6	<0.01 <0.05 <0.05 <0.05	2019
Toral et al. [84]	Normotensive WKY rats	SH rats	N.A.	↓ systolic BP ↓ NADPH oxidase activity ↓ NOX-1 m-RNA ↓ p22^phox^ and p47^phox^ m-RNA	<0.01 <0.01 <0.05 <0.05	2019

**Abbreviations:** ↑, increase; ↓, decrease; B, Bacteroidetes; BP, blood pressure; F, Firmicutes; IL, interleukin; N.A., not applicable; NADPH, Nicotinamide adenosine dinucleotide phosphate; OS, oxidative stress; OSA, obstructive sleep apnea; TNF, tumor necrosis factor; SH, spontaneously hypertensive; WKY, Wistar-Kyoto.

## Data Availability

Non applicable.

## Abbreviations

ACC	American college of cardiology
ADMA	Asymmetric dimethylarginine
AH	Arterial hypertension
AHA	American heart association
B	Bacteroidetes
BP	Blood pressure
CREB	cAMP response element-binding protein
CV	Cardiovascular
DASH	Dietary approaches to stop hypertension
DBP	Diastolic blood pressure
Digitalis-LF	Digitalis-like factor
DOCA	Deoxycorticosterone acetate
ESC	European society of cardiology
ESH	European society of hypertension
F	Firmicutes
Gpr	G protein-coupled receptor
IL	Interleukin
IsoLG	Isolevuglandins
LPS	Lipopolysaccharides
MetS	Metabolic syndrome
NCX1	Sodium–calcium exchanger type 1
NE	Norepinephrine
NO	Nitric oxide
Olfr78	Olfactory receptor 78
RAAS	Renin–angiotensin-aldosterone system
ROS	Reactive oxygen species
SBP	Systolic blood pressure
SCFA	Short chain fatty acid
SHR	Spontaneously hypertensive rat
SMC	Smooth-muscle cells
SNS	Sympathetic nervous system
SS	Salt-sensitive
TMA	Trimethylamine
TMAO	Trimethyilamine N-oxide
TNF	Tumor necrosis factor
